# Glucose metabolism in glioma: an emerging sight with ncRNAs

**DOI:** 10.1186/s12935-024-03499-8

**Published:** 2024-09-13

**Authors:** Jun Rong, Qifu Wang, Tingzheng Li, Jin Qian, Jinchao Cheng

**Affiliations:** 1 Department of Neurosurgery, Xuancheng People’s Hospital, The Affiliated Xuancheng Hospital of Wannan Medical College, Xuancheng, People’s Republic of China; 2 Department of Neurosurgery, The First Affiliated Hospital of Wannan Medical College (Yijishan Hospital), WuHu, People’s Republic of China; 3 Department of Neurosurgery, Xuancheng Central Hospital, Xuancheng, People’s Republic of China

**Keywords:** Glycolysis, Warburg effect, Glioma, ncRNAs

## Abstract

Glioma is a primary brain tumor that grows quickly, has an unfavorable prognosis, and can spread intracerebrally. Glioma cells rely on glucose as the major energy source, and glycolysis plays a critical role in tumorigenesis and progression. Substrate utilization shifts throughout glioma progression to facilitate energy generation and biomass accumulation. This metabolic reprogramming promotes glioma cell proliferation and metastasis and ultimately decreases the efficacy of conventional treatments. Non-coding RNAs (ncRNAs) are involved in several glucose metabolism pathways during tumor initiation and progression. These RNAs influence cell viability and glucose metabolism by modulating the expression of key genes of the glycolytic pathway. They can directly or indirectly affect glycolysis in glioma cells by influencing the transcription and post-transcriptional regulation of oncogenes and suppressor genes. In this review, we discussed the role of ncRNAs in the metabolic reprogramming of glioma cells and tumor microenvironments and their abnormal expression in the glucometabolic pathway in glioma. In addition, we consolidated the existing theoretical knowledge to facilitate the use of this emerging class of biomarkers as biological indicators and potential therapeutic targets for glioma.

## Introduction

Gliomas are the most common form of primary brain tumors with high morbidity and mortality rates [1]. Diagnosis of glioma usually requires a combination of clinical (symptom evaluation), imaging (such as MRI and CT scans), and histologic (such as biopsies or pathology after surgical resection) analysis [2]. In addition, molecular and immunohistochemical analyses may be used to determine the type and grade of gliomas [3]. In fact, glioma cells’ metabolic state has been found to be influenced by a variety of point mutations and copy number changes, which in turn affect tumor growth and patient outcomes. GBM is characterized by a number of common changes, including IDH mutations, EGFR amplification and mutation, PTEN loss, and MGMT promoter mutation. These changes have been associated to enhanced glycolysis, OXPHOS glutamine addiction, and altered lipid metabolism [4]. Adult diffuse gliomas include astrocytoma IDH-mutant (IDH-MUT), oligodendroglioma IDH-MUT and 1p/19q codeleted, and glioblastoma IDH-wild-type (IDH-WT) [5]. The incidence of IDH-MUT in low-grade glioma patients is as high as 80%, while it is almost non-existent in GBM. Patients with IDH-MUT gliomas typically have a longer survival period and better response to chemotherapy and radiation therapy, while wild-type gliomas have relatively poorer outcomes. In glycolysis, IDH-mut are known to shift the metabolic pathway from the typical oxidative decarboxylation of isocitrate to α-ketoglutarate, towards the production of the oncometabolite D-2-hydroxyglutarate. This metabolic reprogramming contributes to the aggressive nature of certain cancers like gliomas by altering the cell’s bioenergetic balance and promoting tumorigenesis [4]. Therefore, the IDH-MUT status has significant clinical significance in the diagnosis and prognostic evaluation of gliomas [5].

The primary treatment for glioma involves surgery, complemented by radiotherapy, chemotherapy, immunotherapy, adjuvant therapy, and other comprehensive treatment modalities [6, 7]. The prognosis depends on several factors, including tumor type, grade, treatment response, and age and overall health of the patient [8]. In general, high-grade gliomas have a poor prognosis, whereas benign gliomas have a good prognosis [9]. Current treatment approaches often fail to provide patients with long-term survival and the best quality of life, and gliomas eventually relapse or progress. However, molecular characterization of gliomas has facilitated the development of targeted therapies [10]. Therefore, it is crucial to address the complexity inherent in glioma biology and further consolidate our understanding of the genesis and developmental mechanisms of glioma to explore new therapeutic strategies.

Metabolism is an important biological process for cell survival, converting nutrients into energy (ATP), redox equivalents (NADPH), and macromolecules (such as lipids) [11]. The altered energy metabolism is one of the hallmarks of cancer cells. It is characterized by a preference for aerobic glycolysis—a process where glucose is converted to pyruvate and subsequently lactic acid—as a means to produce energy regardless of oxygen availability [12]. Compared with anaerobic glycolysis, aerobic glycolysis can produce more ATP in glioma cells for tumor cell proliferation [13]. Abnormal glucose metabolism is clinically used to diagnose cancer and assess tumor response using the radiolabeled glucose analog 18-fluorodeoxyglucose in positron emission tomography [14]. Glucose transporters (GLUTs) [15], kinases (HKs, PFK-1, and PKs) [16], and transcription factors (HIF-1α, c-myc, and p53) [17] are the main regulators of glycolysis. In addition, mTOR, PI3K/AKT, and AMPK signaling are closely associated with glycolysis in gliomas [18].Overall, glycolysis is intricately linked to the activity of glioma cells; therefore, it is a promising target for novel glioma treatment approaches.

**Fig. 1:**
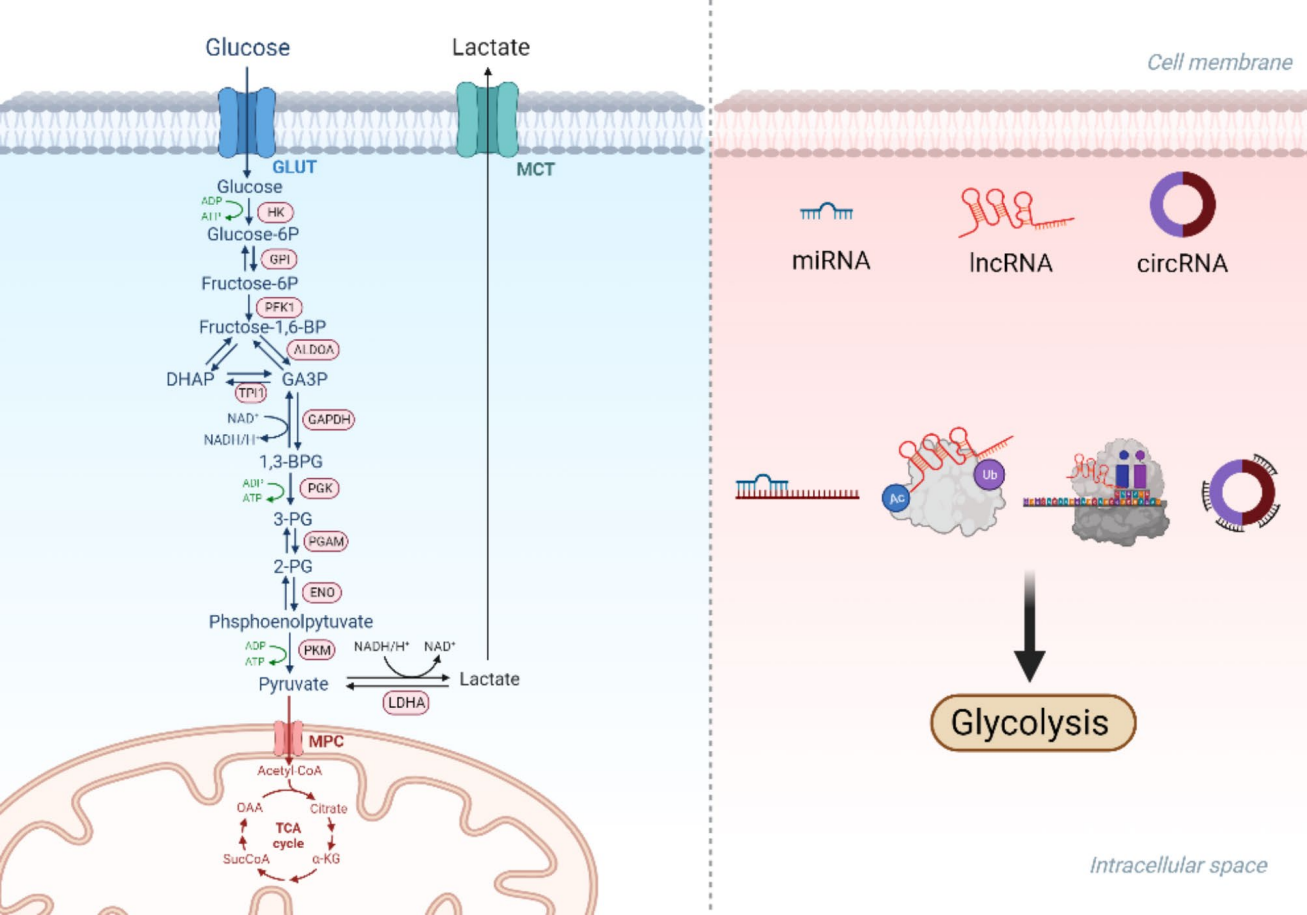
The process of glycolysis and the main mechanisms by which ncRNA affects glycolysis. Glucose enters tumor cells through glucose transporters and undergoes a series of enzymatic reactions to ultimately generate lactate, which is then transported out of the body through lactate transporters. NcRNA can affect the expression of glucose transporters, lactate transporters, glycolytic enzymes, and oncogenes through various mechanisms, thereby affecting the glycolytic process. Created with BioRender.com

Non-coding RNAs (ncRNAs) constitute a distinctive category of RNA transcripts, encompassing over 90% of the human genome. They are categorized into microRNAs (miRNAs), long non-coding RNAs (lncRNAs), and circular RNAs (circRNAs) [19]. NcRNAs are involved in several physiologic processes in gliomas, including tumorigenesis, proliferation, invasion, and metabolism. They regulate the expression of key genes at epigenetic, transcriptional, and post-transcriptional levels (Fig. 1) [20]. Several oncogenes and tumor suppressor genes regulate changes in energy metabolism of gliomas [21]. The influence of ncRNAs on these target genes can promote or inhibit glycolysis (Table 1). Here, we reviewed the mechanisms by which ncRNAs contribute to the regulation of glycolysis in gliomas and elaborated on the possibility of using these ncRNAs for the diagnosis and treatment of gliomas. We clarified the complex interplay between glioma glucose metabolism and ncRNAs and discussed the potential diagnostic targets and therapeutic modalities for glioma management.

**Table 1:** NcRNAs mediating glycolysis in gliomas

ncRNAs	Expression	Target	Role in glycolysis	Actions in gliomas	References
miR-495	↓	GLUT1	Inhibiting	Inhibit proliferation	[28]
miR-3189	↓	GLUT3	Inhibiting	Inhibit tumor growth and induces cell death	[29]
miR-106a	↓	GLUT3	Inhibiting	Inhibit proliferation	[32]
miR-181b	↓	SP1/GLUT1 and PKM2 axis	Inhibiting	Inhibit proliferation	[35]
miR-143	↓	HK2	Inhibiting	Promote GSLC differentiation	[41]
miR-218	↓	Bmi1/HK2 axis	Inhibiting	Inhibit angiogenesis	[42]
miR-128-3p	↓	PDK1	Inhibiting	Inhibit proliferation and induce apoptosis	[46]
miR-1297	↓	KPNA2	Inhibiting	Inhibit proliferation	[49]
miR-124-3p	↓	Pim1	Inhibiting	Inhibit proliferation, invasion and induce apoptosis	[52]
LINC00174	↑	miR-152-3p/GLUT1	Promoting	Promote proliferation, migration and invasion	[61]
lncRNA XIST	↑	miR-126/IRS1/PI3K/Akt/ GLUT1 and GLUT3 axis	Promoting	Promote cell viability, migration, invasion and resistance to apoptosis	[63]
lncRNA DRAIC	↓	NF-κB/GLUT1 axis	Inhibiting	Promote autophagy	[66]
lncRNA ANXA2P2	↑	miR-9/LDHA axis	Promoting	Promote proliferation	[67]
LINC00689	↑	miR-338-3p/PKM2 axis	Promoting	Promote proliferation, migration and invasion	[70]
lncRNA HOTAIR	↑	miR-125/HK2 axis	Promoting	Promote proliferation and inhibit TMZ resistance	[72]
LINC00470	↑	FUS/AKT/HK1 axis	Inhibiting	Inhibit autophagy	[74]
lncRNA ZBED3-AS1	↓	SPI1/THBD axis	Inhibiting	Inhibit TMZ resistance, viability and mobility	[78]
lncRNA JPX	↑	FTO/PDK1 axis	Promoting	Promote proliferation, TMZ chemoresistance, anti-apoptosis and DNA damage repair	[79]
LncRNA MDHDH	↓	PSMA1/MDH2 axis	Inhibiting	Inhibit proliferation, migration and invasion	[84]
lncRNA NEAT1	↑	PGK1	Promoting	Promote proliferation	[86]
lncRNA LINK-A	↑	LDHA	Promoting	Inhibit proliferation, migration and invasion	[88]
lncRNA SNHG5	↑	miR-205/E2F3 axis	Promoting	Promote migration and invasion	[90]
lncRNA SNHG9	↑	miR-199a-5p/Wnt2 axis	Promoting	Promote proliferation	[91]
lncRNA SNHG14	↑	IRF6/PKM2 and GLUT1 axis	Promoting	Promote proliferation	[92]
LINC01138	↑	miR-375/SP1 axis	Promoting	Promote proliferation	[95]
lncRNA PCED1B-AS1	↑	HIF-1α	Promoting	Promote proliferation	[102]
lncRNA NKILA	↑	NF-κB/HIF-1α axis	Promoting	Promote angiogenesis	[105]
LINC02774	↓	RP58/PHD3/HIF-1α axis	Inhibiting	Inhibit proliferation, migration and invasion	[108]
circZNF609	↑	miR-378b/GLUT1 axis	Promoting	Promote proliferation	[115]
circKIF4A	↑	miR-335-5p/ALDOA axis	Promoting	Promote proliferation and TMZ resistance	[119]
circHEATR5B	↓	JMJD5/PKM2 axis	Inhibiting	Inhibit proliferation	[121]
circSOBP	↓	TKFC	Inhibiting	Inhibit proliferation, migration and invasion	[122]
circ_0072083	↑	miR-5-1252p/ ALKBH5/NANOG axis	Promoting	Promote TMZ resistance, proliferation, migration and invasion	[125]
circPITX1	↑	miR-329-3p/NEK2 axis	Promoting	Promote proliferation and radioresistance	[127]
circNFIX	↑	miR-378e/RPN2 axis	Promoting	Promote migration and invasion	[131]

This table summed up the dysregulation of miRNAs, lncRNAs, and circRNAs involved in the glycolysis in gliomas and their targets.

## MiRNAs regulating glucose metabolism in glioma

**Fig. 2:**
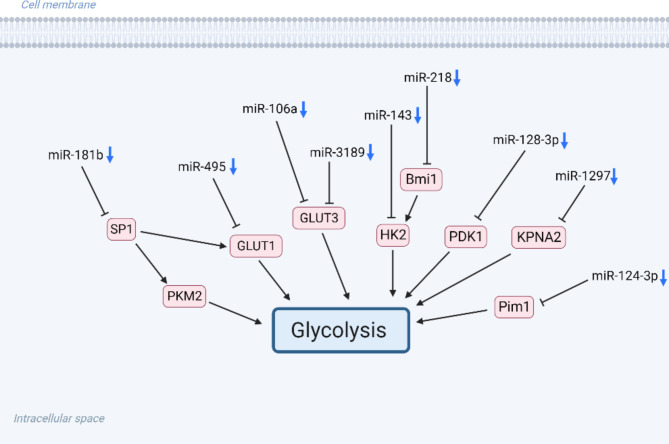
MiRNAs regulates glycolysis in gliomas. MiRNAs mainly regulates glycolysis by binding to the 3’UTR of target genes and inhibiting their expression. Created with BioRender.com

MiRNA is a type of single-stranded ncRNA molecule, typically between 21 and 23 nucleotides in length. MiRNAs regulate gene expression at the translational level [22, 23]. Certain miRNAs function as tumor suppressor genes and inhibit the proliferation, invasion, and metastasis of tumor cells. However, some miRNAs may act as tumor-promoting genes and promote tumor growth and metastasis [24]. Abnormal expression of miRNAs rrinfluences various biological processes in glioma, including glycolysis (Fig. 2) [25]. The research on the mechanism of action and clinical application of miRNAs in glioma will help understand the molecular mechanism of glioma development and subsequently develop new therapeutic targets and strategies [26].

### MiRNAs affecting glucose transporters

MiRNAs can directly target the 3′ untranslated region (3′UTR) of mRNA and inhibit the target gene expression in gliomas, thereby influencing the tumor progression [27]. For example, miR-495 can directly target the 3′UTR of glucose transporter GLUT1 and inhibit its expression, leading to increased glucose uptake and lactate production in glioma cells [28]. Kwak et al. found that histone deacetylase 2 can inhibit the expression of miR-3189, thereby blocking the inhibitory effect of miR-3189 on GLUT3. Therefore, targeting histone deacetylase 2 can inhibit glucose metabolism in GBM to exert antitumor effects [29]. MiR-106a is downregulated in various tumor types and functions as a tumor suppressor [30, 31]. Dai et al. discovered that the expression of miR-106a was notably diminished in the GBM tissue. Furthermore, patients showing reduced miR-106a expression levels had lower survival rates. MiR-106a can target GLUT3 to decrease glucose uptake in GBM cells, thereby inhibiting cell proliferation [32]. MiRNAs can also affect the expression of downstream glucose transporters by directly targeting transcription factors [33, 34]. MiR-181b directly targets the transcription factor SP1 and suppresses its expression, thereby inhibiting the expression of several downstream genes including GLUT1 and pyruvate kinase M2 (PKM2). Consequently, glucose metabolism and proliferation capabilities of GBM cells are impaired [35].

### MiRNAs influencing glycolytic enzymes

MiRNAs can bind to the mRNAs of glycolysis-related genes, resulting in the degradation of mRNAs or inhibition of translation [36, 37]. This process affects the expression levels of glycolysis-related genes. Hexokinase 2 (HK2) is the initial rate-limiting enzyme in the glycolytic pathway, and its abnormal expression in gliomas promotes malignant progression [38–40]. MiR-143 can suppress glycolysis by directly targeting HK2, thereby promoting the differentiation of glioblastoma stem-like cells (GSLCs). Moreover, the combined application of miR-143 and a commonly used glycolytic inhibitor 2-DG exerts a synergistic antitumor effect on GSLCs. Therefore, miR-143 is a potential therapeutic target for glioblastoma treatment [41]. MiR-218 is another miRNA that regulates HK2 expression. Liu et al. observed that miR-218 downregulates HK2 expression by binding to the 3′UTR of Bmi1, thereby inhibiting the malignant progression of glioma cells [42].

Pyruvate dehydrogenase kinase 1 (PDK1) is crucial in the transition from glycolysis to the tricarboxylic acid cycle. Its upregulation in tumors shifts oxidative phosphorylation towards the Warburg effect [43–45]. MiR-128-3p targets PDK1, leading to decreased glycolysis levels and dysfunction of mitochondria in glioma cells. Therefore, targeting PDK1 with miR-128-3p represents a potential therapeutic approach for glioma treatment [46].

### MiRNAs regulating oncogenes

MiR-1297 is dysregulated in various cancers and targets different oncogenes or tumor suppressor genes to exert its biological effects [47, 48]. MiR-1297 is significantly downregulated in GBM, and its overexpression can significantly inhibit the proliferation and glycolysis of GBM cells. It can directly target the 3′UTR of KPNA2 (a key regulatory factor in glycolysis) to inhibit glycolysis [49]. Pim1, a kinase belonging to the oncogenic Pim kinase family, regulates cell proliferation, cell cycle, apoptosis, and metabolism in various human cancers [50, 51]. MiR-124-3p targets the 3′UTR of PIM1 and decreases its mRNA and protein expression levels. This decrease inhibits aerobic glycolysis and proliferation and invasion of astrocytoma cells while promoting cell apoptosis [52].

Taken together, miRNAs play a crucial regulatory role in the initiation, progression, and treatment of tumors. Research on miRNAs helps understand the molecular mechanisms underlying tumors and identify novel targets and strategies for tumor diagnosis, treatment, and prognosis evaluation.

## LncRNAs regulating glucose metabolism in glioma

**Fig. 3:**
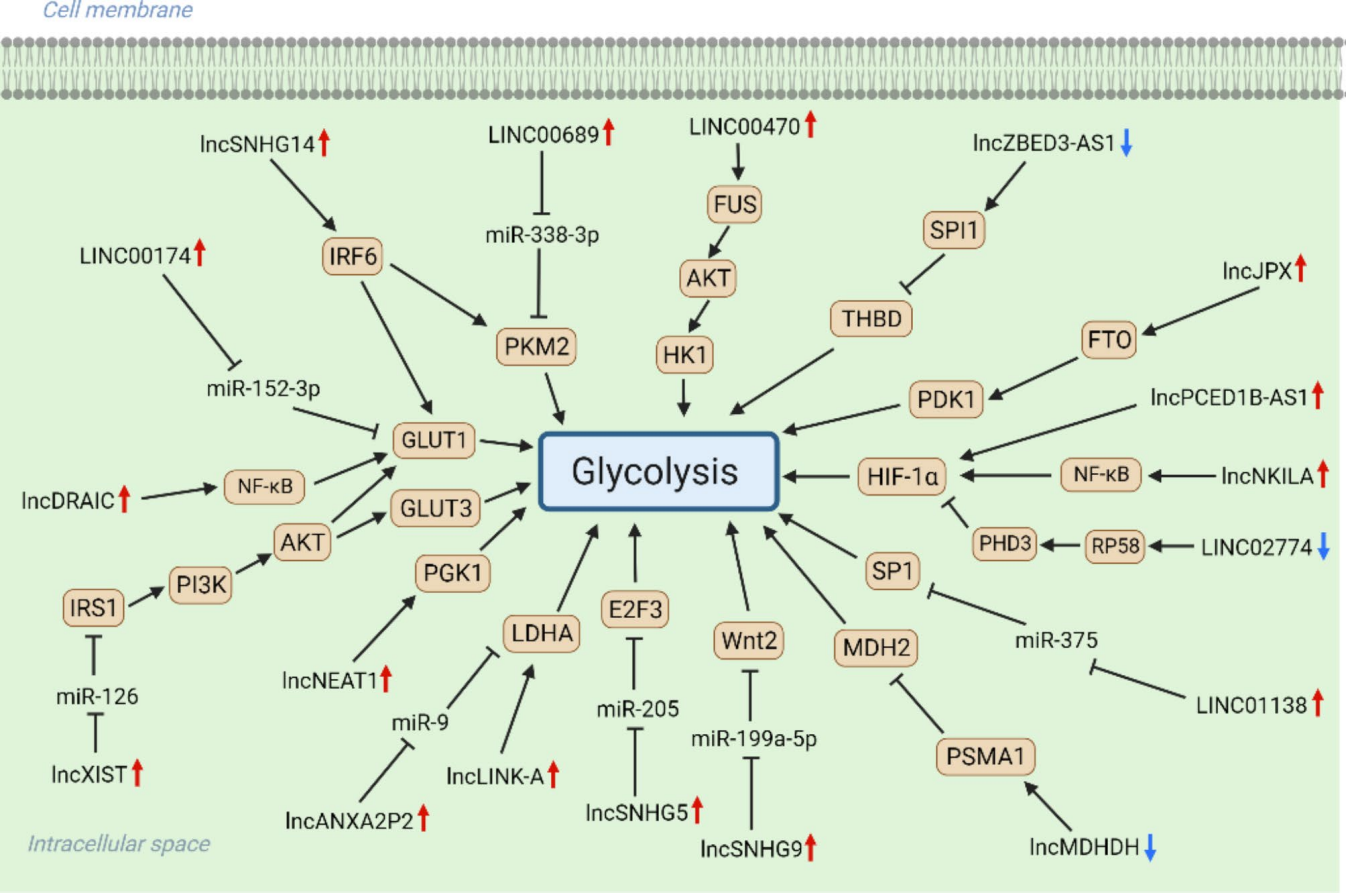
LncRNAs regulates glycolysis in gliomas. The regulatory mechanisms of lncRNAs on glycolysis include ceRNA, direct binding with target proteins to affect their stability, and binding with transcription factors to affect the transcription of target genes. Created with BioRender.com

LncRNAs are single-stranded RNAs with more than 200 nucleotides in length [53]. LncRNAs cannot encode proteins but are widely present in various cells. The expression of multiple lncRNAs is dysregulated in tumors [54, 55]. These abnormally expressed lncRNAs can affect cellular signaling, gene expression, and epigenetic regulation through different mechanisms, thereby promoting or inhibiting tumor development [56]. LncRNAs have garnered widespread attention in tumor research because of their important role in tumors. Glycolysis-associated lncRNAs can serve as predictive markers for the prognosis, immune infiltration, and epithelial-to-mesenchymal transition status of glioma patients [57, 58]. LncRNAs may affect glycolysis in gliomas in various ways, thereby affecting tumor progression (Fig. 3). These findings offer novel insights and targets for further research on glioma.

### LncRNAs affecting glucose transporters

LncRNAs can function as competitive endogenous RNAs (ceRNAs) and act as a sponge for binding miRNAs. This interaction blocks the regulatory effect of miRNA on the downstream genes, thereby modulating gene expression [59, 60]. For instance, LINC00174 can upregulate GLUT1 by competitively binding to miR-152-3p in glioma cells. This interaction promotes glycolysis and proliferation, migration, and invasion of glioma cells [61]. LncRNA X-inactive specific transcript (XIST) mediates the transcriptional silencing of genes located on the X chromosome. Dysregulated lncRNA XIST expression is associated with an increased risk of various cancers [62]. LncRNA XIST acts as a ceRNA of miR-126, thereby regulating the IRS1/PI3K/Akt pathway. This regulatory mechanism results in elevated expression of GLUT1 and GLUT3, consequently promoting glycolysis and malignant progression in glioma cells [63]. NF-κB, a transcription factor, regulates the expression of various metabolic enzymes including key enzymes of the glycolytic pathway [64, 65]. LncRNA DRAIC inhibits NF-κB, resulting in decreased expression of GLUT1. This inhibition ultimately leads to reduced glycolysis levels and induces autophagy [66].

### LncRNA regulates glycolysis by influencing glycolytic enzymes

The expression of lncRNA Annexin A2 Pseudogene 2 (ANXA2P2) is markedly upregulated in glioma tissues and cells. Conversely, its knockdown significantly inhibits the proliferation of glioma cells and aerobic glycolysis in them. LncRNA ANXA2P2 can bind to miR-9, which, in turn, directly targets the 3′UTR of lactate dehydrogenase A (LDHA) and inhibits its expression. This regulatory mechanism modulates the Warburg effect and influences the proliferation and apoptosis of glioblastoma cells [67].

PKM2 is the final rate-limiting enzyme in glycolysis, and its heightened activity is correlated with the growth, proliferation, and metastasis of tumor cells [68, 69]. The expression of LINC00689 is upregulated in glioma tissues and cell lines, and it is associated with a malignant phenotype and poor prognosis in patients with glioma. LINC00689 promotes the expression of PKM2 by directly interacting with miR-338-3p, thereby promoting glycolysis. This interaction facilitates the proliferation, migration, and invasion of glioma cells [70].

The expression level of homologous cassette transcript antisense intergenic RNA (HOTAIR) is upregulated in various cancers, and it is associated with the malignancy and prognosis of tumors [71]. Zhang et al. reported that HOTAIR can target miR-125 to promote the expression of HK2, thereby facilitating glycolysis and proliferation of GBM cells and inhibiting temozolomide (TMZ)-induced cell apoptosis [72]. The direct binding of lncRNA to proteins enables the regulation of protein activity, stability, and subcellular localization [73]. This interaction affects the function of proteins, thereby regulating cellular processes [56]. For instance, LINC00470 binds to fused in sarcoma (FUS) protein in glioma cells and sequesters FUS in the cytoplasm to enhance AKT activity and inhibit the ubiquitination of HK1, consequently regulating glycolysis and suppressing autophagy [74].

High levels of glycolysis in glioma cells are correlated with resistance to chemotherapy drugs [15, 75]. In addition, lncRNAs can mediate treatment resistance in glioma cells by influencing glycolysis. TMZ is a commonly used chemotherapy drug for the treatment of malignant gliomas [76, 77]. It inhibits the growth and spread of tumor cells by interfering with DNA synthesis and repair. LncRNA ZBED3-AS1 is notably downregulated in TMZ-resistant GBM tissues and cell lines. ZBED3-AS1 binds to the Spi-1 oncogene and blocks the transcriptional activity of its downstream gene thrombomodulin, thereby inhibiting glycolysis and TMZ resistance in glioma cells [78]. LncRNA just proximal to the X-inactive specific transcript (JPX) is significantly elevated in GBM tissues and cell lines, and its high expression is significantly associated with poor prognosis. LncRNA JPX enhances FTO-mediated demethylation of PDK1 mRNA by forming complexes with this mRNA, thereby promoting its stability and expression. This action ultimately facilitates aerobic glycolysis and TMZ resistance in GBM cells [79].

Ubiquitination is an important intracellular protein modification process that regulates protein stability, activity, and localization [80–82]. Some lncRNAs can interact with ubiquitinase or ubiquitination substrate proteins and modulate ubiquitination [83]. The expression of a novel lncRNA MDHDH is markedly reduced in patients with GBM. It acts as a molecular scaffold by directly binding to malate dehydrogenase 2 and 20 S proteasome core subunit α Type 1 (PSMA1). This binding accelerates the degradation of ubiquitinated malate dehydrogenase 2, consequently impeding glycolysis and inhibiting the proliferation, migration, and invasion of glioma cells [84].

LncRNA nuclear paraspeckle assembly transcript 1 (NEAT1) is involved in tumor cell growth and metabolic reprogramming and is significantly overexpressed in gliomas [85]. Overexpression of NEAT1 stabilizes PGK1 by direct interaction, consequently promoting glycolysis and tumor progression in gliomas [86].

Long intergenic non-coding RNA for kinase activation (LINK-A) is an intergenic lncRNA that activates normoxic HIF-1α signaling in triple-negative breast cancer [87]. It is markedly upregulated in glioma cells and promotes glycolysis and cell proliferation through positive regulation of LDHA [88].

### LncRNA regulating oncogenes

LncRNA small nuclear RNA host genes (SNHGs) regulate gene transcription and play a pivotal role in the development and progression of cancer. These genes can function as either oncogenes or tumor inhibitors [89]. Li et al. reported that lncRNA SNHG5 is highly expressed in gliomas, and its expression correlates with glucose uptake, migration, and invasion of gliomas. It acts as a sponge for miR-205 to regulate downstream E2F transcription factor 3 expression, thereby promoting glucose uptake and tumor progression in glioma [90]. SNHG9 is highly expressed in glioblastoma, and its overexpression promotes cell growth and aerobic glycolysis through the miR-199a-5p/Wnt2 axis [91]. LncRNA SNHG14 can interact with the mRNA of transcription factor IRF6 and induce its degradation, relieving the transcriptional inhibition of PKM2 and GLUT1 by IRF6. The resulting increase in glycolysis promotes the proliferation of glioma cells [92].

SP1 is a transcription factor that regulates the transcription of multiple genes encoding glucose transporters and glycolytic enzymes [93, 94]. Therefore, SP1 plays a crucial role in regulating glycolysis. Xu et al. discovered that LINC01138 can promote glycolysis and proliferation of glioma cells through the miR-375/SP1 axis [95]. Therefore, it may be targeted to regulate glycolysis in glioma cells.

Hypoxia often occurs due to insufficient angiogenesis and abnormal metabolism in tumor tissues [96]. Hypoxia can promote the activation of glycolytic pathways, thereby providing energy and biosynthetic substances to tumor cells [97, 98]. Hypoxia and the hypoxia-inducible factor (HIF)-1α promote tumor progression in gliomas by inducing anaerobic glycolysis [99]. Multiple lncRNAs in gliomas can act as upstream regulatory factors for HIF-1α to regulate HIF-1α-induced glycolysis [100, 101]. LncRNA PCED1B-AS1 can directly interact with the 5′UTR of HIF-1α mRNA, enhancing HIF-1α translation. The increase in HIF-1α protein levels promotes the Warburg effect and tumorigenesis in glioma cells [102]. NF-κB interacting lncRNA (NKILA) interacts with NF-κB to inhibit the NF-κB pathway and exert antitumor effects [103, 104]. However, Zheng et al. found that NKILA is significantly upregulated in gliomas, and it can enhance the expression of HIF-1α and the activity of hypoxia-induced pathways. Consequently, NKILA promotes the Warburg effect and angiogenesis in gliomas [105].

LncRNAs can bind to transcription factors and regulate their activity, thereby affecting the expression of specific genes [106, 107]. The nuclear lncRNA LINC02774 is significantly downregulated in glioma and is negatively correlated with malignancy. It interacts with transcription factor RP58 and affects the RP58/PHD3/HIF-1α axis to regulate glycolysis in glioma cells [108]. LncRNA Highly Upregulated in Liver Cancer (HULC) was initially identified for its high expression and carcinogenic effects in liver cancer [109]. HULC promotes glycolysis and the stemness of GSCs by regulating the FOXM1/AGR2/HIF-1α axis, consequently exacerbating the occurrence and development of glioma [110]. Taken together, lncRNAs are the potential biomarkers and therapeutic targets for the diagnosis, treatment, and prognosis evaluation of glioma.

However, several challenges persist in the current landscape of lncRNA research. First, investigations into the mechanistic actions of lncRNAs predominantly revolve around their role as miRNA sponges within the lncRNA-miRNA-mRNA regulatory axis and other potential mechanisms remain unexplored. Secondly, several authors have elaborated on the downstream target genes of lncRNAs, whereas the upstream regulatory factors influencing lncRNA expression have not been discussed. It is imperative to address these gaps in knowledge to elucidate the complex mechanisms underlying lncRNA involvement in glioma glycolysis.

## CircRNAs regulating glucose metabolism in glioma

**Fig. 4:**
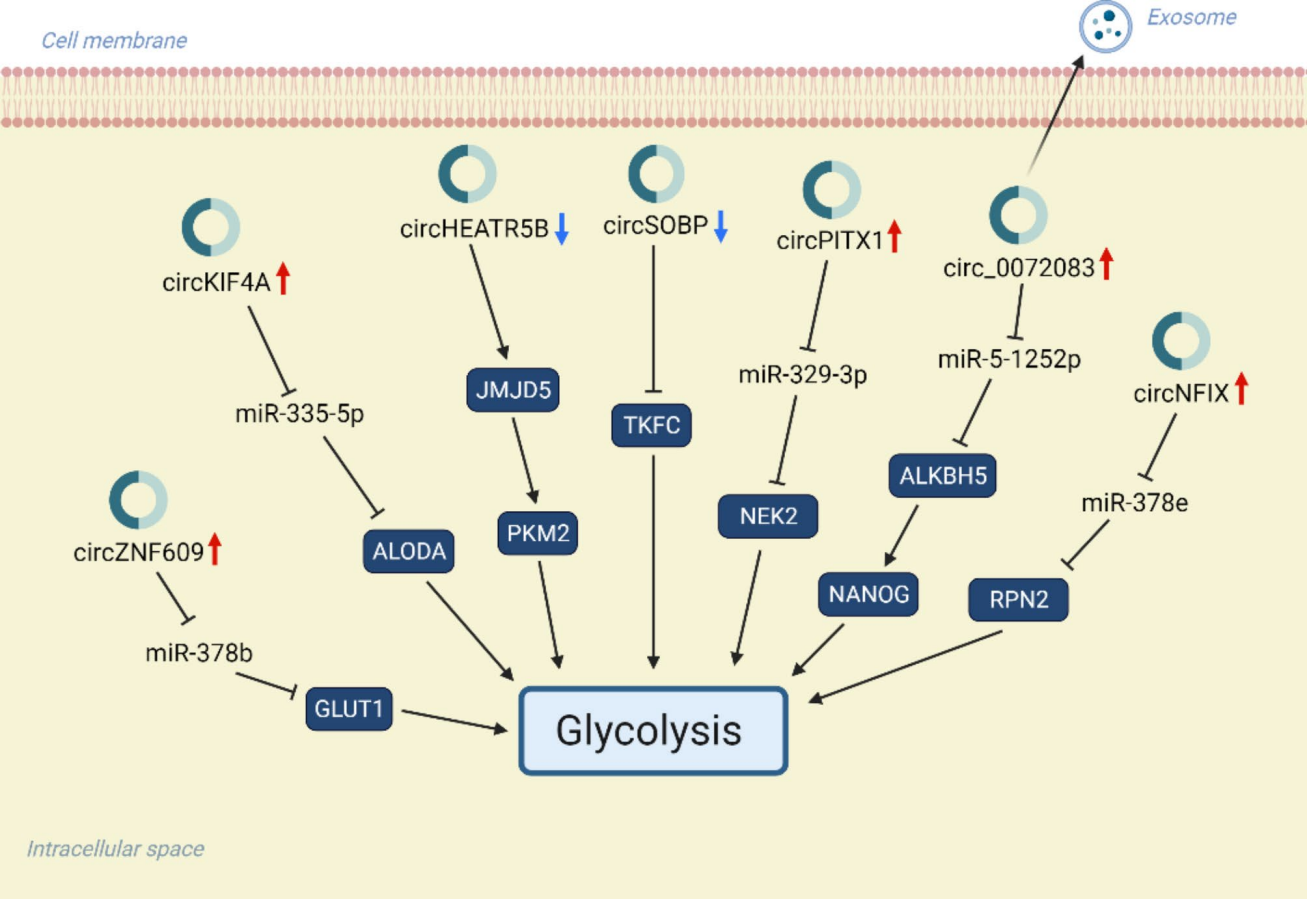
circRNAs regulates glycolysis in gliomas. The mechanism by which CircRNAs affect glycolysis is similar to that of lncRNA, but the unique circular structure of circRNA provides better stability and weaker immunogenicity, making it a huge advantage in diagnosis and treatment. Created with BioRender.com

CircRNA is a novel class of circular non-coding RNAs generated through the process of reverse splicing of precursor mRNA (pre-mRNA) [111]. CircRNAs regulate various cellular functions and participate in tumor progression through different mechanisms [112]. CircRNAs are not degraded by RNA exonucleases due to their closed circular structure. Therefore, they are notably stable and less susceptible to degradation. CircRNAs act as “sponges” for miRNAs and modulate their activity by competitive binding, thereby influencing the regulatory effect of miRNAs on their target genes. In addition, certain circRNAs can directly interact with proteins and affect protein function and stability. The dysregulation of circRNA in gliomas mediates the process of glycolysis (Fig. 4).

### CircRNAs affecting glucose transporters

CircRNA zinc finger protein 609 (ZNF609) originates from exon 2 of the ZNF609 gene and is abnormally upregulated in various tumors. Its increased expression promotes tumor cell proliferation, migration, and invasion [113]. It is significantly upregulated in glioma cells, where it contributes to the malignant progression of tumors [114]. Mechanically, circRNA ZNF609 enhances the expression of glucose transporter SLC2A1 by inhibiting miR-378b, thereby promoting glycolysis and cell survival in glioma [115].

### CircRNAs influencing glycolytic enzymes

CircKIF4A can promote tumor progression by acting as ceRNA [116–118]. It is notably upregulated in glioma tissues and cell lines, where it binds to miR-335-5p to regulate the glycolytic regulatory enzyme ALDOA. This interaction promotes glycolysis, proliferation, and TMZ resistance in glioma cells [119].

In addition, certain circRNAs can encode peptides or proteins to participate in the progression of tumors [120]. Song et al. identified circRNA HEATR5B, which encodes a novel protein named HEATR5B-881aa. Overexpression of HEATR5B-881aa inhibited aerobic glycolysis in GBM cells and limited their proliferation. HEATR5B-881aa directly interacts with Jumonji C-domain containing 5 (JMJD5) and decreases its stability by phosphorylating S361. The decreased expression of JMJD5 enhances the activity of PKM2 and suppresses glycolysis in GBM cells [121]. Mu et al. discovered that circSOBP can directly interact with triokinase/FMN cyclase protein in glioma cells. This interaction inhibits glycolysis and enhances MDA5-mediated immune response, thereby suppressing the progression of glioma. Therefore, circSOBP is a promising therapeutic target to inhibit glioma glycolysis [122].

### CircRNAs regulating oncogenes

Extracellular vesicles are small secretory vesicles containing bioactive substances such as proteins, nucleic acids, and lipids [123, 124]. Ding et al. revealed that the Warburg effect enhances the release of extracellular vesicle circ_0072083 in glioma-resistant cells. This circRNA blocks ALKBH5-mediated demethylation and decreases NANOG expression by targeting miR-5-1252p. This mechanism promotes malignant phenotypes, such as drug resistance, proliferation, migration, and invasion, in glioma cells [125]. CircPITX1 is upregulated in glioblastoma [126]. It can upregulate NEK2 by targeting miR-329-3p, thereby promoting glycolysis, radiation resistance, cell survival, and clonogenic ability of glioma cells in vitro. Moreover, it can also facilitate tumor growth in vivo [127]. CircNFIX is highly expressed and has a carcinogenic role in various cancers, including lung cancer [128], hepatocellular carcinoma [129], and ovarian cancer [130]. It is also upregulated in gliomas and significantly associated with poor prognosis in patients. CircNFIX can bind to miR-378e through the ceRNA mechanism, leading to the upregulation of downstream gene RPN2. This upregulation promotes glycolysis in glioma cells and enhances their migration, and invasion [131]. CircRNAs hold significant promise in glioma research, and further investigations into their role will furnish novel insights into the diagnosis, treatment, and prognosis evaluation of glioma.

## Conclusions and future perspectives

According to studies, ncRNAs can be found in biological fluids like blood and cerebrospinal fluid. They also exhibit distinct expression patterns in a variety of physiological and pathological states, which makes them a viable option for use as biomarkers for the diagnosis, prognosis, and tracking of different human malignant tumor treatments [132]. Dysregulation of circulating miRNAs has been discovered in several clinical investigations in GBM patients, offering prospective biomarkers for GBM diagnosis and surveillance [133]. For example, the glycolysis related miRNA miR-124-3p mentioned earlier has been found to be associated with increased expression in serum exosomes and the progression of high grade gliomas (HGG) [134]. Following surgery, there was a significant drop in the expression of miR-124-3p in the serum exosomes of HGG patients. This suggests that ncRNA associated to glycolysis may be used as a biomarker for clinical assessment of early tumor progression [135].

NcRNAs are critical regulatory elements in tumor metabolism [136]. They modulate the expression of target genes, including those encoding glucose metabolism enzymes, lipid synthase, and amino acid metabolism enzymes. This regulatory activity influences the metabolic traits of tumor cells and their development and progression [137]. Lipid nanoparticles (LNPs) are lipid-based carriers that have been approved by the FDA to transport RNA to cells. Examples of these applications include COVID-19 vaccinations and therapies for hereditary transthyretin-mediated amyloidosis. They shield nucleic acids, facilitate their uptake into cells, and are being investigated for targeted cancer treatments. Developments in this field are opening up new therapeutic options for cancer [138]. Therefore, regulating glycolysis through delivery or targeting ncRNAs has become a promising therapeutic approach for GBM.

Currently, various RNA-based therapies have been approved for the clinical treatment of non-tumor diseases [19, 139]. When it comes to treating disorders at the molecular level, ncRNA-based therapies have an advantage over standard medications in that they can precisely target specific gene expression. Furthermore, ncRNAs are adaptable tools for treating complicated disorders like cancer and GBM because they have the ability to control a wide range of biological processes [140]. Therapeutic methods based on siRNA and miRNA have advanced into clinical trials, showcasing their potential value in the treatment of tumors [141]. To investigate the potential of RNA-based treatments, there are also ongoing clinical trials in the field of brain disorders. A mutation in exon 1 of the HTT gene called CAG amplification results in the generation of mutant Huntington’s (mHTT) protein, which causes Huntington’s disease (HD), an autosomal dominant genetic illness [142]. Targeting the pathophysiology of HD, the application of antisense oligonucleotides (ASO) and miRNA targeting HTT to lower mHTT levels has demonstrated encouraging potential in the realm of HD treatment. Recombinant adeno-associated viral serotype 5 (rAAV5)-miHTT, a microRNA-based adenovirus vector carrying targeted human HTT, is being tested for safety and tolerability in patients with early-stage HD as part of the ongoing AMT-130 Phase I/II clinical trial (NCT04120493, NCT05243017), and there is initial evidence of both clinical and functional benefits [143]. This approach is based on earlier research on the safety and effectiveness of miHTT therapy in preclinical animals, where it has been demonstrated that rAAV5-miHTT may be able to slow down HD neurodegeneration [144–146]. WVE-003 is an allele selective ASO targeting rs362273 (SNP3) located on the mHTT gene, and preclinical studies suggest that WVE-003 may reduce mHTT in patients while maintaining wtHTT expression [147]. As part of the treatment of HD patients, a multicenter, randomised, double-blind, placebo-controlled Phase 1b/2a research (NCT05032196) is now being conducted to assess the safety, tolerability, pharmacokinetics, and pharmacodynamics of intrathecal injection of WVE-003 [147]. Thus far, one clinical trial (NCT03020017) has been carried out to administer siRNA targeting oncogene Bcl2L12 to patients suffering from glioblastoma or gliosarcoma utilizing metal nanocarriers (NU-0129). Following an intravenous injection, glioma cells’ uptake of NU-0129 was linked to a drop in the expression of the tumor-associated Bcl2L12 protein, indicating the potential of nanocarriers and siRNA as targeted agents for brain invasion [148].

However, ncRNA-based treatment methods have several limitations. The delivery of ncRNA is one of the main obstacles in GBM treatment. Their transportation in the body becomes challenging due to their molecular size and structural characteristics, impeding their ability to cross the blood–brain barrier or maintain stability in the body [149]. Secondly, the insufficient targeting ability of ncRNAs limits their application. NcRNA-based therapy may induce non-specific gene silencing, which may limit treatment efficacy. Nucleic acid aptamers are molecules that can specifically bind to target nucleic acid sequences. They are composed of nucleic acid molecules or proteins and can interact with target nucleic acid sequences through base pairing or specific binding methods [150]. Nucleic acid aptamers can serve as carriers for ncRNA, achieving precise delivery by specifically binding to targets [151]. Preclinical studies have affirmed the potential of targeting ncRNA-mediated glycolysis in the treatment of GBM. However, clinical studies are warranted to validate the efficacy and safety of ncRNA-based therapies in clinical applications. Therefore, future studies should further elucidate the ncRNA-mediated regulation of glycolysis in GBM. Clinical applications of ncRNA-based therapies in GBM depend on designing more effective delivery systems, and personalized strategies, overcoming biological barriers, reducing side effects, and strengthening clinical research and translation.

## Data Availability

No datasets were generated or analysed during the current study.

## Abbreviations

GBM: glioblastoma multiforme
GLUTs: Glucose transporters
ncRNAs: Non-coding RNAs
miRNAs: microRNAs
lncRNAs: long non-coding RNAs
circRNAs: circular RNAs
3′UTR: 3′ untranslated region
PKM2: pyruvate kinase M2
HK2: Hexokinase 2
GSLCs: glioblastoma stem-like cells
PDK1: Pyruvate dehydrogenase kinase 1
ceRNAs: competitive endogenous RNAs
XIST: X-inactive specific transcript
ANXA2P2: Annexin A2 Pseudogene 2
LDHA: lactate dehydrogenase A
HOTAIR: homologous cassette transcript antisense intergenic RNA
TMZ: temozolomide
FUS: fused in sarcoma
JPX: just proximal to the X-inactive specific transcript
PSMA1: 20 S proteasome core subunit α Type 1
NEAT1: nuclear paraspeckle assembly transcript 1
LINK-A: Long intergenic non-coding RNA for kinase activation
SNHGs: small nuclear RNA host genes
HIF: hypoxia-inducible factor
NKILA: NF-κB interacting lncRNA
HULC: Highly Upregulated in Liver Cancer
pre-mRNA: precursor mRNA
ZNF609: zinc finger protein 609
JMJD5: Jumonji C-domain containing 5
HGG: high grade gliomas
LNPs: Lipid nanoparticles
HD: Huntington’s disease
ASO: antisense oligonucleotides
rAAV5: recombinant adeno-associated viral serotype 5
